# Characteristics of Trocar Site Hernia after Laparoscopic Cholecystectomy

**DOI:** 10.1038/s41598-020-59721-w

**Published:** 2020-02-18

**Authors:** Mohammad Nibih Nofal, Ali Jad Yousef, Farouq Fakhri Hamdan, Ahmad Hisham Oudat

**Affiliations:** 1 General Surgery and Anesthesia Department, College of Medicine, Mutah University, Mu’tah, Jordan; 2 General Surgery Department, Al-Bashir Hospital, Ministry of Health/ Amman- Jordan, Amman, Jordan; 3 Medical student, College of Medicine, Mutah University, Mu’tah, Jordan

**Keywords:** Diseases, Gastroenterology

## Abstract

Trocar site hernia (TSH) is an incisional hernia occurring at the trocar insertion sites after different types of laparoscopic surgeries. The aim of this study is to present characteristics of patient and surgery series with trocar site hernia after laparoscopic cholecystectomy. A 2930 consecutive patients underwent laparoscopic cholecystectomy in two major university- affiliated hospitals from April 2014 to March 2018 and the patient followed up for variable periods of time. Retrospective medical chart review to study trocar site hernia including patient, operation, instruments, and pathologic characteristics described. Six patients had trocar site hernia (incidence 0.20%), the hernias occurred mostly at the umbilical port site after using 10 mm trocar. Risk factors included mainly obesity, female gender and use of 10 mm trocars at midline sites. TSH is more described. It occurs mostly at the umbilical port site. Major risk factors include obesity, diabetes mellitus, lengthy procedure, extension of entry site, and wound infection. Closure of fascial defect is supposed to reduce the incidence despite weak evidence.

## Introduction

Laparoscopic cholecystectomy (LC) was performed for the first time by Erich Mühe of Bo¨blingen, Germany on September 12, 1985^1^. It is now the standards surgical treatment for gallbladder stone disease (GBSD) and stood the test of time regarding reduced postoperative pain, decreased hospital stay, earlier return to work, and much more cosmetic scars. On the other hand, LC appeared progressively to have unprecedented complications such as complications related to the pneumoperitoneum and laparoscopic instruments in addition to the classical complications encountered in the open surgery such as bleeding, bile leak, and common bile duct injury.

The trocar site hernia was reported for the first time in the late 1960s of the last century in a series of patients underwent laparoscopic gynecologic procedures^2^. The first case of TSH after LC was reported by Maio *et al*. in the USA in 1991in which they described the use of CT scan to diagnose the hernia^3^. Consequently, variable several studies were published addressing laparoscopic surgery complications including trocar site hernia. In this research, we present our observations regarding TSH after 2930 cases of LC.

## Methods

This is a retrospective medical records review of 2930 consecutive patients underwent LC for GBSD in two Mutah University College of Medicine –affiliated hospitals: Al Bashir Hospital in Amman, and Al Karak Governmental Teaching Hospital, Karak, Jordan from April 2014 to March 2019. The research methods were carried out in accordance with Scientific Research Committee in the College of Medicine in Mutah University guidelines and approved by the Institutional Review Boards (IRB) Committee on Ethics in Scientific Research in the College of Medicine in Mutah University: All patients signed informed consent to use medical information for scientific research on admission. The patient’s exclusion criteria were age younger than 18 years, conversion to open cholecystectomy, pre-existing umbilical hernia, immunodeficiency disorders, re-operation due to other complication of LC than TSH, and patients lost to follow up. Studied variables included patient related factors: age, sex, weight, co morbidities, hernia site and gall bladder pathology; surgery related factors: elective versus emergency, trocar size used at hernia site, entry technique, intraoperative bleeding, operation time, and closure of fascial layers.

The LC surgeries were performed by three surgical teams. In all, but one patient, the closed technique was used to establish pneumoperitoneum, bladeless and bladed trocars used. A transverse, infra-umbilical, 1–2 cm long skin incision was made and a −10 mm reusable trocar was inserted into the peritoneal via a vertical incision along the linea alba and the peritoneum. After inserting the first trocar and establishing pneumoperitoneum of 14 mmHg, two more plastic bladed trocars, 10 and 5 mm, were introduced under direct vision through incisions just below the xiphoid and at the right midclavicular line, 4–6 cm below the costal margin, respectively. A fourth 5 mm trocar was inserted at the right anterior axillary line, 6–8 cm below the costal margin. After complete dissection of the gallbladder, it was retrieved through the infra-umbilical incision. The fascia at the infra-umbilical incision was sutured with interrupted Prolen sutures No. 0 in the patients presented with acute cholecystitis. The fascia was sutured at sites of 10 mm ports with Prolen or Vicryl No. 0 but not at sites of 5 mm ports. Operation time, pathology reports, and hernia occurrence recorded for all patients. The TSH was diagnosed clinically in all patients.

## Results

From April 2014 to March 2018, a total number of 2930 patients underwent LC for GBSD in two university – affiliated hospitals by three surgical teams. The operative procedure was the standard LC procedure. Six patient developed TSH at different periods of follow up that represents an incidence of 0.2%. No one of the patient was old. The median age of the patients was 41.8 years(31–55years). The male: female ratio was 1:2. Two female patients were hypertensive, and one of them, the patient who presented acutely, had also sleep apnea (SA). Obesity, defined as body mass index (BMI) > 30 found in 66.7% of patients. The patient demographics and operative findings are presented in Table 1.

**Table 1:** Patient demographics and some operative findings and details.

Pt. No.	Age	Sex	Obesity	Morbidities	Elective Vs Emergency	Operative time	Single /Multiple stones	Other GB Pathology	Trocar size	Entry technique	Hernia Site	Sutured fascia
1	M	50	Yes	None	Elective	20 min	Multiple	None	10 mm	Closed	Epigastric	Yes
2	F	31	No	None	Elective	30 min	Multiple	None	10 mm	Closed	Umbilical	Yes
3	F	55	No	HT	Elective	45 min	Multiple	None	10 mm	Open	Umbilical	Yes
4	F	40	Yes	None	Elective	30 min	Multiple	None	10 mm	Closed	Umbilical	Yes
5	M	32	Yes	None	Elective	40 min	Multiple	None	10 mm	Closed	Epigastric	Yes
6	F	43	Yes	HT SA	Emergency	2 hours	Single large stone	Acute Cholecystitis	10 mm	Closed	Umbilical	Yes

HT: Hypertension, SA: Sleep apnea.

The mean operative time was 41.8 minutes (range 20–120 minutes). The longest duration was for the emergency LC for acute cholycystitis. There was no need to extend the fascial opening to extract the gallbladder in any patient. None of the LC was associated with intra operative bleeding or bile duct injuries. All patients had multiple small GBS, except the patient with acute cholecystitis who had single large stone. No tumors or polyps found the GB specimens. The earliest patient to present with TSH in this series was the patient who presented acutely and had the longest operative time. The remaining patients presented within one year of surgery. No patient presented with evisceration. One third of the hernias occurred in the epigastric port site, the remaining occurred in the umbilical port site. Most of them presented with painless slowly growing mass at the trocar site, not associated with acute or obstructive symptoms. The content of the hernia sac was omentum. The TSH was repaired electively with simple anatomic repair.

## Discussion

LC is the most common laparoscopic procedure performed worldwide, and like other types of laparoscopic surgery, it is responsible for the development of a new type of incisional hernia occurring at the insertion site of the trocar, the so-called trocar site hernia (TSH) as defined by Crist and Gadacz^4^. However, the term ‘port site hernia” used to describe the same clinical situation in other reports. The classification of TSH by Tonouchi *et al*. introduced in 2004 classified TSH into three types: (i) the early onset type characterized by anterior–posterior fascial plane and peritoneum dehiscence; (ii)the late onset type occurring months or years post operatively and characterized by anterior and posterior fascia plane dehiscence whereas the peritoneum constitutes the hernia sac; and (iii) a special type of hernia, occurring immediately post operatively and characterized by dehiscence of the whole abdominal wall and visceral protrusion^5^. The third type of Tonouchi *et al*. classification is really a true evisceration in the classical surgical terms in which the incisional hernia is differentiated from evisceration by the presence of intact skin and hernia sac. Interestingly, the first case of TSH reported in 1968 by Fear *et al*. was of this type.

The development of incisional hernia after classical major laparotomy is a well-known complication. However, the development of incisional hernia after laparoscopic surgery is much less common with a prevalence reported to be less than 1% in different series [6, 7, and 8]. The available data is scant and came mostly from retrospective studies with ill- defined follow-up periods and study cut points. The incidence of TSH after LC is controversial; earlier reports described this complication as extremely rare, while many recent publications mentioned it as very common. Table 2 summarizes various incidence results from different series worldwide over the last two decades.

**Table 2:** The incidence of TSH in different studies worldwide.

Study (Ref)	Year	Country	TSH/Total LC	TSH%
Voyles^6^	1991	USA	1/500	0.20%
Larson^20^	1992	USA	3/1983	0.15%
Baird^21^	1992	USA	1/800	0.13
Azurin^9^	1995	USA	10/1300	0.77%
Ahmad^22^	1997	USA	11/1300	0.84%
Nassar^23^	1997	UK	16/870	1.83%
Sanz-Lopez^24^	1999	Spain	2/123	1.62%
Al-Haijar^12^	2002	Romania	10/1453	0.68%
Uslu^10^	2007	Turkey	40/766	5.2%
Comajuncosa**s**^7^	2014	Spain	57/220	25.9%
Chatzimavroudis^11^	2017	Greece	11/1127	0.94%

The incidence in this series was similar to that of Voyles *et al*. series^6^. The highest incidence reported is 25.9% in Comomajuncosas *et al*. series^7^ with a conclusion that the incidence is actually was higher (47.4%) due to the presence of asymptomatic TSH cases detected after thorough physical examination. We expect that TSH incidence will be higher if patients are not lost to follow up and if imaging modalities are used to investigate equivocal clinical findings.

TSH occurs primarily at the umbilical trocar site because most trocars used in the umbilicus are large^8^, or probably umbilical hernia was there before laparoscopic operation^9^. In this report, two-third of cases occurred at the umbilical trocar site.

Different risk factors for TSH such as age, female gender, diabetes, obesity (BMI > 30), type of cholecystitis, extension of umbilical incision, trocar size, trocar site, wound infection, duration of surgery, preexisting hernia, non closure of trocars site defect, poor muscle relaxation during reversal of anesthesia before trocar removal were analyzed using univariate and multivariate statistical analysis in variable retrospective and prospective cohorts. All of the previous factors were found significant on univariate analysis. However, on multivariate analysis the list of significance was shortened to advancing age (>70), BMI > 30, duration of surgery, diabetes mellitus, incision enlargement, and wound infection as detailed in Table 3^7,10,11^.

**Table 3:** Multivariate analysis of risk factors for TSH.

Risk Factor	Study	p- Value	Odds ratio	Confidence Interval
Obesity	Uslu *et al*.^10^	0.000	34.526	N/A
	Comajuncosas *et al*.^7^	0.009	2.71	1.28–5.75
	Chatzimavroudis *et al*.^11^	< 0.01	5.81	1.98–17.73
Duration of Surgery	Uslu *et al*.^10^	0.000	66.414	
Age	Uslu *et al*.^10^	0.003	5.106	
Diabetes Mellitus	Comajuncosas *et al*.^7^	0.0038	2.79	1.05–7.37
Incision Enlargement	Comajuncosas *et al*.^7^	< 0.001	14.17	3.61–55.51
wound infection	Comajuncosas *et al*.^7^	< 0.001	5.62	2.35 to 13.42

Obesity causes chronic increase in intra abdominal pressure and weakness of the abdominal wall musculature. However, we believe that the hernia will be more obvious if the patient loses weight due to the reduced buttressing effect of the subcutaneous fat. Although no statistical inference can be drawn from this series due to the small sample size, nonetheless, few benchmarks deserve attention such as the most of the cases are in females, and most the patients are obese, and all the hernias appeared in the midline trocars at trocar size of 10 mm. Most of the cases were chronic cholecystitis due to multiple GBS, and the only case with single stone presented with acute cholycystitis. The presence of malnutrition or cholecystitis was suggested as possible risk factors for TSH in some reports without statistical analysis^8,12^. Two of our patients were hypertensive and no one proved diabetic at time of surgery or during follow up. The hernia content can be omentum and/ or bowel hence the variation in the clinical manifestations from asymptomatic and discovered by ultrasound or CT scanning to mild pain and slowly growing mass indicating omental entrapment, whilst severe pain and change of skin color over the mass indicates early complication such as omental infarction or bowel incarceration and obstruction^13^ that requires emergency surgery in about 15% of patients^14^. In this series the entire patients present with early pathologic stage without complication. No intestine was entrapped in the hernia sac. The clinical onset of TSH can be early as one month post operative or years later. The surgical treatment depends on the presentation and intraoperative findings. Closure of the fascia was suggested to reduce the incidence of TSH. However, it is still a controversial issue. In this series the fascia was closed at 10 mm or more port sites. Some authors suggest the routine closure of fascia entrance of 10 mm or larger trocars^15,16^ while others find this unnecessary step^17,18^. New trend suggest sclosing the fascia with mesh in certain high- risk patients^19^.

## Conclusion

The incidence of TSH is rising probably due to increased use of laparoscopic techniques and increased awareness and methods of TSH diagnosis. Although the current evidence is weak regarding the causative risk factors, nonetheless, it seems obesity is a dominating risk factor and patients should be warned about the occurrence of TSH preoperatively. Closure of 10 mm or larger trocars sites seems more appropriate in preventing TSH although the evidence is lacking.

## Data Availability

The materials of his study are available.
